# Tris DBA palladium is highly effective against growth and metastasis of pancreatic cancer in an orthotopic model

**DOI:** 10.18632/oncotarget.10514

**Published:** 2016-07-09

**Authors:** Begoña Díaz, Katherine T. Ostapoff, Jason E. Toombs, Jason Lo, Michael Y. Bonner, Adam Curatolo, Volkan Adsay, Rolf A. Brekken, Jack L. Arbiser

**Affiliations:** ^1^ Department of Dermatology, Emory University School of Medicine, Atlanta, GA, USA; ^2^ Atlanta Veterans Administration Medical Center, Atlanta, GA, USA; ^3^ Winship Cancer Institute, Atlanta, GA, USA; ^4^ Tumor Microenvironment and Metastasis Program, Cancer Center, Sanford Burnham Prebys Medical Discovery Institute, La Jolla, CA, USA; ^5^ Division of Medical Oncology and Hematology, Los Angeles Biomedical Research Institute at Harbor-UCLA Medical Center, Torrance, CA, USA; ^6^ Department of Surgery, Hamon Center for Therapeutic Oncology Research, UT Southwestern Medical Center, Dallas, TX, USA; ^7^ Department of Pharmacology, Hamon Center for Therapeutic Oncology Research, UT Southwestern Medical Center, Dallas, TX, USA; ^8^ Vascular Biology Program, Department of Surgery Children's Hospital Boston, Boston, MA, USA; ^9^ Karp Family Research Laboratories, Harvard Medical School, Boston, MA, USA; ^10^ Department of Pathology and Laboratory Medicine, Emory University School of Medicine, Atlanta, GA, USA

**Keywords:** pancreatic cancer, tris DBA palladium, NMT1, metastasis, chemotaxis

## Abstract

Pancreatic carcinoma ranks among the most lethal of human cancers. Besides late detection, other factors contribute to its lethality, including a high degree of chemoresistance, invasion, and distant metastases. Currently, the mainstay of therapy involves resection of local disease in a minority of patients (Whipple procedure) and systemic gemcitabine. While systemic chemotherapy has some benefit, even with optimal treatment, the five year survival after diagnosis is dismal. Thus, treatment of pancreatic carcinoma remains a tremendous unmet need.

The organometallic compound tris DBA palladium is a potent inhibitor of N-myristoyltransferase 1 (NMT1), an enzyme that catalyzes the transfer of myristate to protein substrates. This compound is highly effective *in vivo* against murine models of melanoma with both mutant and wild type b-RAF genotypes. Based upon the signaling similarities between melanoma and pancreatic carcinoma, we evaluated the efficacy of tris DBA palladium *in vitro* and *in vivo* against pancreatic carcinoma. We found that tris DBA palladium decreased proliferation and colony formation of pancreatic cancer cells *in vitro*. In an orthotopic mouse model, tris DBA palladium was highly active in inhibiting growth, ascites production, and distant metastases *in vivo*. Furthermore, tris DBA palladium impaired chemotaxis and inhibited cilia formation in Pan02 cells in a NMT1-dependent manner. We propose that NMT1 is a novel regulator of cilia formation and tris DBA palladium a novel inhibitor of cilia formation and metastasis in pancreatic cancer. Thus, further evaluation of tris DBA palladium for the treatment of pancreatic cancer is warranted.

## INTRODUCTION

Carcinoma of the exocrine pancreas remains among one of the deadliest solid tumors in humans. Factors that contribute to the high degree of mortality are late detection, when distant metastases are present, anatomical location making definitive surgery difficult, and advanced age. Over 90% of pancreatic adenocarcinomas exhibit mutations in Kras, which is currently undruggable [1, 2]. Thus, surgery and radiation alone are insufficient to control the disease. Chemotherapy is commonly used, with gemcitabine and folfirinox being the mainstays of therapy [3].

Additional studies have attempted to increase the success of therapy of pancreatic adenocarcinoma. The use of hedgehog inhibitors to block signaling and desmoplasia resulted in decreased desmoplasia, but with no clinical benefit [4]. Thus, novel therapies are urgently needed for this common and lethal cancer [5].

Tris DBA palladium (Tris DBA thereinafter) is a small molecule that we found to be a novel inhibitor of N-myristoyltransferase 1 (NMT1) [6]. NMT1 is responsible for myristoylation of multiple proteins, including the src family kinase (SFK) proteins [7, 8]. Lack of myristoylation compromises the function of target proteins, as it prevents their localization to cell membranes [9]. In this study, we tested the possibility that tris DBA could have anti-tumor effects in pancreatic adenocarcinoma. Here we demonstrate that tris DBA exhibits a potent antiproliferative effect on pancreatic cancer cell lines *in vitro*. Furthermore, in an orthotopic pancreatic xenograft mouse model, tris DBA decreased primary tumor growth and the occurrence of distant metastasis. To understand the mechanism by which tris DBA reduced tumor metastasis, we examined the effect of this compound on cancer cell migration and cilia formation. Cilia mediate directed cell migration and their presence in human pancreatic carcinoma has been recently associated with poor prognosis [10]. We propose that tris DBA should be studied further as a potential novel treatment for pancreatic carcinoma with non-overlapping mechanisms of action to other drugs.

## RESULTS

Tris DBA is highly active *in vivo* against melanoma driven by different genetic alterations [6]. Given that melanoma and pancreatic carcinoma share some common genetic and signaling features, including loss of p16Ink4a, predisposing to melanoma and pancreatic carcinoma, we evaluated the effect of tris DBA treatment in pancreatic carcinoma.

### Tris DBA decreased pancreatic cancer cell growth *in vitro*

**Figure 1:**
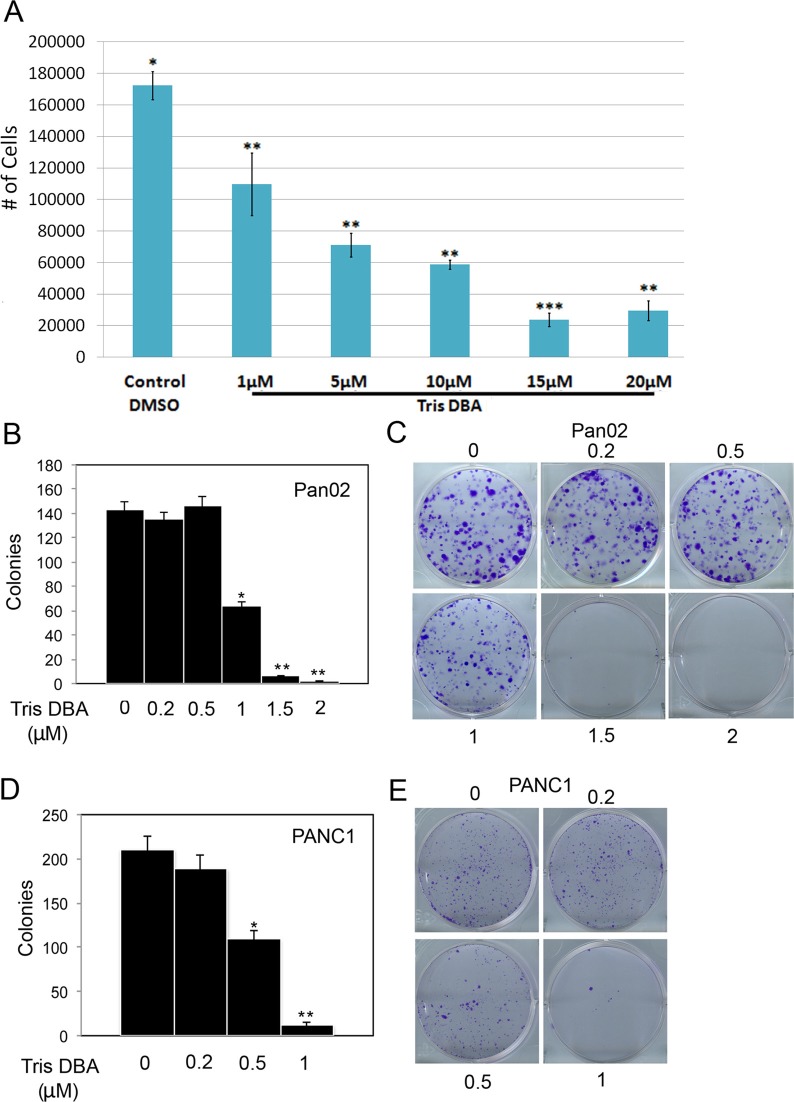
Tris DBA decreases proliferation and colony forming ability of pancreatic carcinoma cells (**A**) Pan02 cells were treated for 24 h with the indicated concentrations of tris DBA or control vehicle (DMSO). Cells were counted 24 h after treatment and average of quadruplicate experiments ± S.D. represented. (*) *p* < 0.05; (**) *p* < 0.005 (***) *p* < 0.0005 (Student's *t* test) (**B**) Pan02 were plated at low density and treated 24 h after seeding with the indicated concentrations of tris DBA or vehicle control. Number of colonies visualized with crystal violet 6 days after treatment was quantified and represented as average ± S.D. of triplicate experiments. (*) *p* < 0.001; (**) *p* < 0.00001 (Student's *t* test). (**C**) Representative images of crystal violet-stained colonies after treatment of Pan02 cells with the indicated concentrations of tris DBA. (**D**) PANC1 were plated at low density and treated 24 h after seeding with the indicated concentrations of tris DBA or vehicle control. Number of colonies visualized with crystal violet 6 days after treatment was quantified and represented as average ± S.D. of triplicate experiments. (*) *p* < 0.001; (**) *p* < 0.0001 (Student's *t* test). (**E**) Representative images of crystal violet-stained colonies after treatment of PANC1 cells with the indicated concentrations of tris DBA.

We tested the effect of tris DBA in the murine pancreatic carcinoma cell line Pan02 and found that it was highly effective in reducing proliferation after 24 h of treatment in a dose-dependent fashion (Figure 1A). To analyze the effect of longer treatments, we performed colony forming assays using different concentrations of the compound. Tris DBA significantly reduced colony formation in Pan02 cells (Figure 1B, 1C), suggesting impaired proliferation and/or survival of cells upon treatment. Tris DBA was also highly effective in reducing colony formation in the human pancreatic cell line PANC1 at 0.5 μM (Figure 1D, 1E). In Pan02 cells, tris DBA treatment decreased the phosphorylation of Akt and p42/44 MAPK at approximately 10 μM, whereas phosphorylation of S6 kinase and FoxM1 were unaffected (Supplementary Figure 1). A significant inhibitory effect of the compound in cell growth occurred at a much lower compound concentration (1 μM) than the one needed to inhibit signaling through Akt and p42/44 MAPK (10 μM). Therefore, it is unlikely that the effect of tris DBA in proliferation and colony formation can be explained by alterations in the MAPK or Akt signaling pathways.

### Tris DBA decreased pancreatic tumor growth and metastasis in an orthotopic mouse model

**Figure 2:**
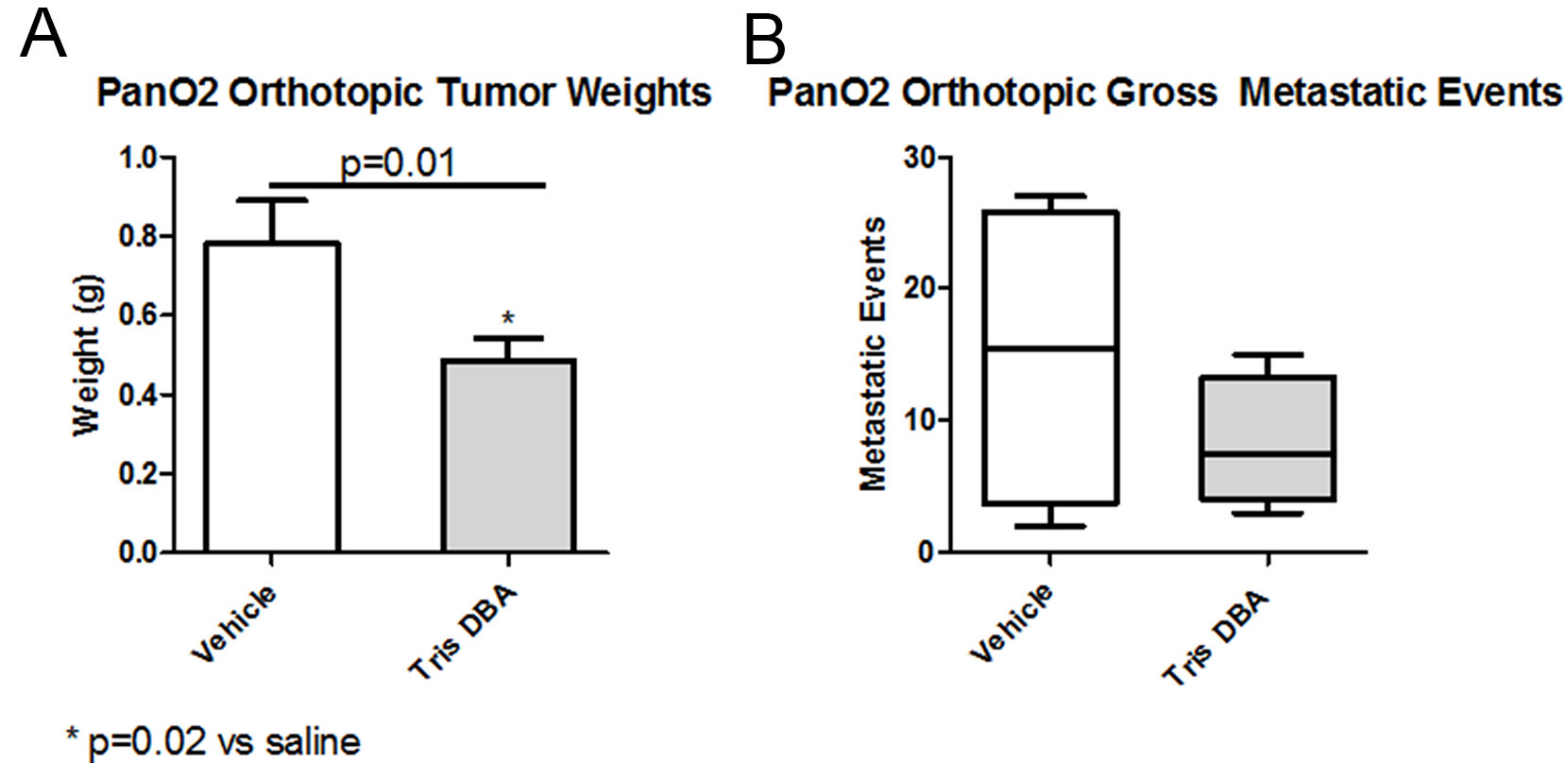
Tris DBA is as efficacious against primary tumor growth and metastasis 6-8 week old C57bl/6 mice, in a group of five for vehicle control and a group of seven for tris DBA, were injected orthotopically with 5 × 10^5^ Pan02 cells. One week post injection, they receive vehicle (coconut oil), tris-DBA 6.4 mg/kg, all three times weekly. (**A**) At sacrifice, tumors were removed and their weight measured. Data shown are mean +/− SEM from a single experiment. ^*^p < 0.05, One way ANOVA with Tukey's MCT. (**B**) Gross liver metastatic events were counted at sacrifice, results were not significant (*p* = 0.34).

To determine whether tris DBA demonstrates efficacy against pancreatic carcinoma *in vivo*, Pan02 cells were orthotopically transplanted into mice. The mice were then randomized to vehicle control or tris DBA. Tris DBA treatment led to significant decreases in tumor weight (Figure 2A) and a reduction in metastatic events (Figure 2B) compared to control-treated animals. No histological differences were observed between the control and treated groups (Supplementary Figure 2).

### Tris DBA impaired chemotactic cell migration in pancreatic cancer cells *in vitro*

**Figure 3:**
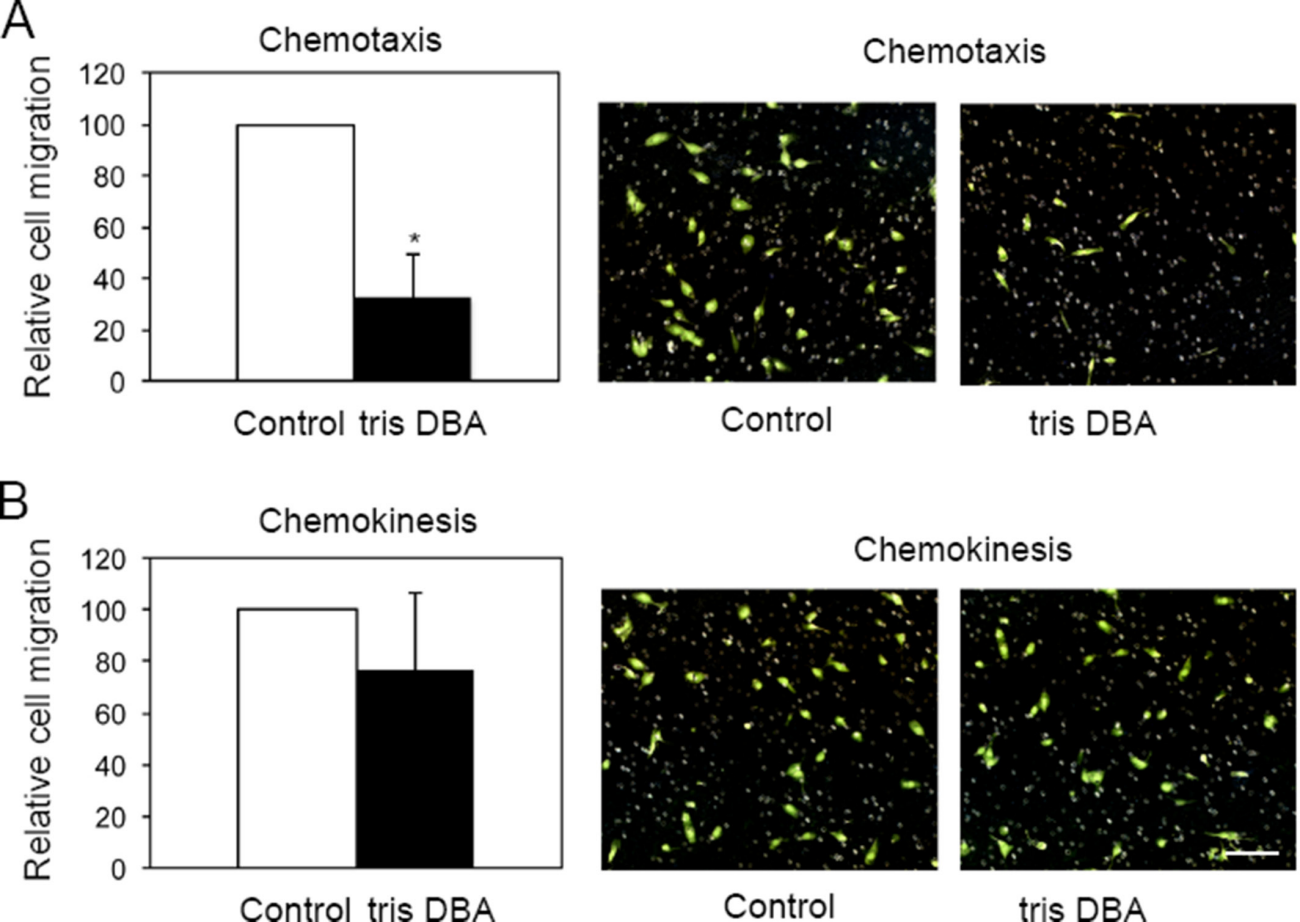
Tris-DBA impairs chemotaxis but not chemokinesis in Pan02 cells Pan02 were assayed for chemotaxis and chemokinesis in the presence or absence of tris-DBA (1mM) using *in vitro* trans-well assays. For chemotaxis (**A**) cells were plated in medium containing 1% FBS in the presence or absence of compound. Lower chambers were filled with medium containing 10% FBS. Cell migration was assessed after 6 hours by counting crystal violet-stained cells under the microscope. Graph represents average number of migrated cells normalized to non-treated control, from three independent experiments. Bars, S.D. (*) *p* = 0.9 × 10^−5^ (Student's *t*-test). For chemokinesis (**B**), the assay was performed under the same conditions with the exception that cells were plated in medium containing 10% FBS. Representative pictures of filters stained with crystal violet are shown at the right of each graph. For clarity, color images were inverted using Adobe Photoshop. Bar, 20 μm.

The striking metastasis inhibitory effect of tris DBA *in vivo* prompted us to analyze whether this drug directly targeted metastasis or whether its effect on metastasis was an indirect consequence of decreased primary tumor volume. Since the migratory ability of cancer cells is associated with its metastatic capacity we investigated the effects of tris DBA in pancreatic cancer cell migration. Chemokinesis (cell migration that is independent of a gradient of soluble factors) and chemotaxis (cell migration induced by a gradient of soluble factors), were modeled *in vitro* using trans-well migration assays. For chemokinesis, cells were plated in the upper chamber in medium containing 10% FBS whereas for chemotaxis assays, cells were plated in the upper chamber in medium containing 1% FBS. In all cases, the lower chambers were filled with medium containing 10% FBS. Interestingly, treatment with tris DBA significantly decreased the ability of Pan02 cells to migrate in the presence (Figure 3A), but not in the absence (Figure 3B), of a FBS gradient. This suggested that chemotaxis but not chemokinesis, was impaired by drug treatment. To verify that impaired attachment or decreased survival of treated Pan02 cells in 1% versus 10% FBS was not accounting for the differences in trans-well migration, we performed a parallel assay in regular growth plates. Control or tris DBA-treated Pan02 cells were plated in the presence of 1% or 10% FBS for 6 h. Cell number was quantified by colorimetry after crystal violet staining and no differences were found across treatments (Supplementary Figure 3). This indicated that the presence of a serum gradient, rather than the final concentration of serum in the plating medium, impacted the migratory ability of Pan02 cells in the presence of tris DBA. We concluded that *in vitro* chemotaxis but not chemokinesis of Pan02 cells was affected by tris DBA treatment.

Additionally, we did not observe differences in the number or size of focal adhesions (as analyzed by FAK staining) after compound treatment (Supplementary Figure 3). This also suggests that the intrinsic ability of cells to migrate may not be affected by the compound. We conclude that tris DBA impaired the ability of cells to sense a gradient of soluble factors, which is a known trigger of cell migration.

### Tris DBA reduces cilia formation in both Pan02 and PANC1 cells

**Figure 4:**
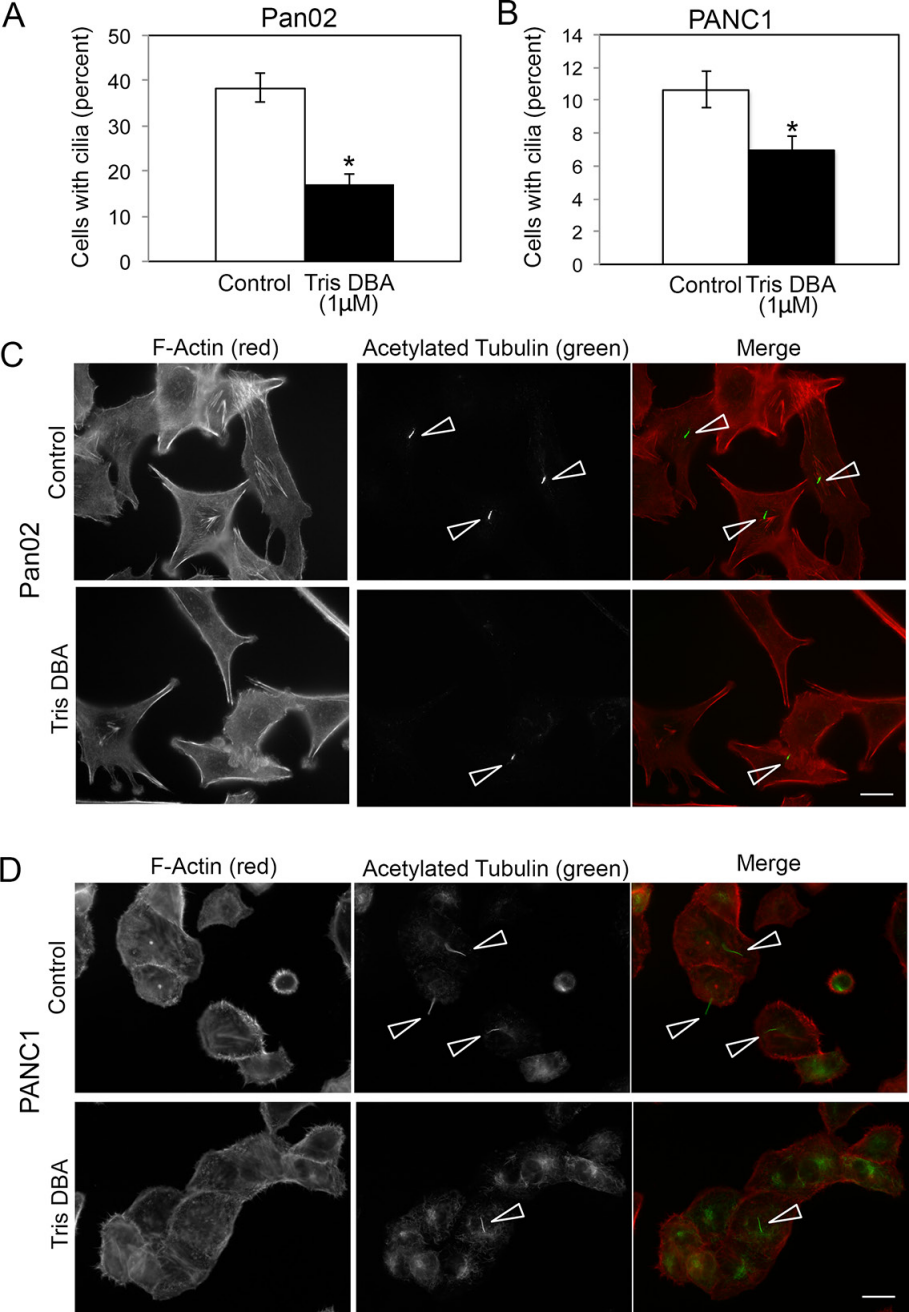
Cilia formation is disrupted by tris-DBA treatment in pancreatic cancer cells Pan02 (**A**) or PANC1 (B) cells were cultured in the presence or absence of tris-DBA (1 mM) for 6 h and processed for detection of cilia by acetylated tubulin staining. Percent of cells forming cilia was quantified in at least 200 cells per condition in three independent experiments. The graphs show average ± S.D. for the percent of cells forming cilia in a representative experiment. (*) *p* < 0.0005 (Student's *t*-test) in (A) and (*) *p* < 0.001 (Student's *t*-test) in (**B**). (**C**) Representative images of cilia in Pan02 cells treated with tris DBA (1 μM) or vehicle control. (**D**) Representative images of cilia in PANC1 cells treated with tris-DBA (1 μM) or vehicle control. Arrowheads, cilia. Bars, 5 μm.

Because cilia have been associated with cellular sensing of gradients of diffusible signals [11, 12], we tested whether tris DBA affected cilia formation in Pan02 cells. To identify cilia, we used an antibody against the well-characterized cilia marker acetylated tubulin. Interestingly, treatment of Pan02 cells with tris DBA caused a significant decrease in the percent of cells forming cilia (Figure 4A, 4C). The effect of tris DBA in cilia formation was not cell-type dependent, as the compound also decreased cilia formation in the human pancreatic cancer cell line PANC1 (Figure 4B, 4D).

Taken together, our data suggest that tris DBA impaired chemotaxis of Pan02 cells by decreasing cilia formation and affecting the ability of cells to sense a gradient of chemotactic factors. The inhibitory effect of tris DBA on chemotaxis and cilia formation suggests a specific effect of this compound in metastasis *in vivo.*

**Figure 5:**
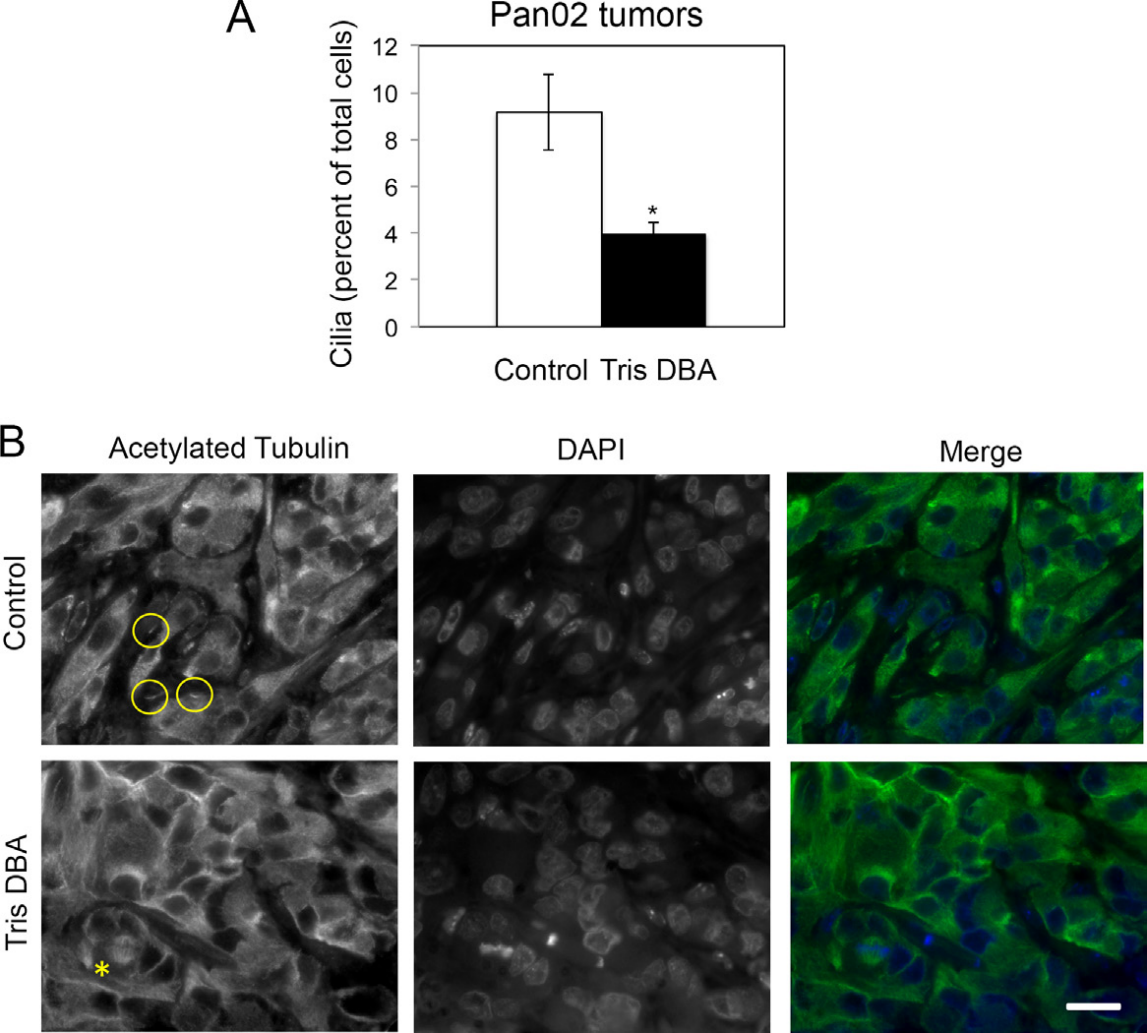
Tris DBA treatment decreases cilia formation in Pan02 orthotopic tumors (**A**) Percent of cells forming cilia in tumor sections from control and tris DBA-treated mice was quantified in 15 randomly chosen fields representing at least 500 cells per condition. The graph show average ± S.E.M. for the percent of cells forming cilia. (*) *p* < 0.005 (Student's *t*-test). (**B**) Representative images of acetylated tubulin-stained tumor sections from the indicated groups. Circles indicate cilia and asterisk indicates a mitotic spindle (positive control for the staining). Bar, 2 μm.

To investigate whether treatment with tris DBA affected cilia formation *in vivo*, we analyzed cilia formation in primary tumors from mice treated with vehicle control or tris-DBA. We stained tumor sections for the cilia marker acetylated tubulin and quantified cilia formation (Figure 5A). The detection of mitotic spindles was used as a positive control for antibody performance (Figure 5B). Interestingly, we observed a significant decrease in the presence of cilia in tumors from tris DBA treated mice (Figure 5A, 5B), suggesting that tris DBA decreases pancreatic tumor cilia formation *in vivo*.

**Figure 6:**
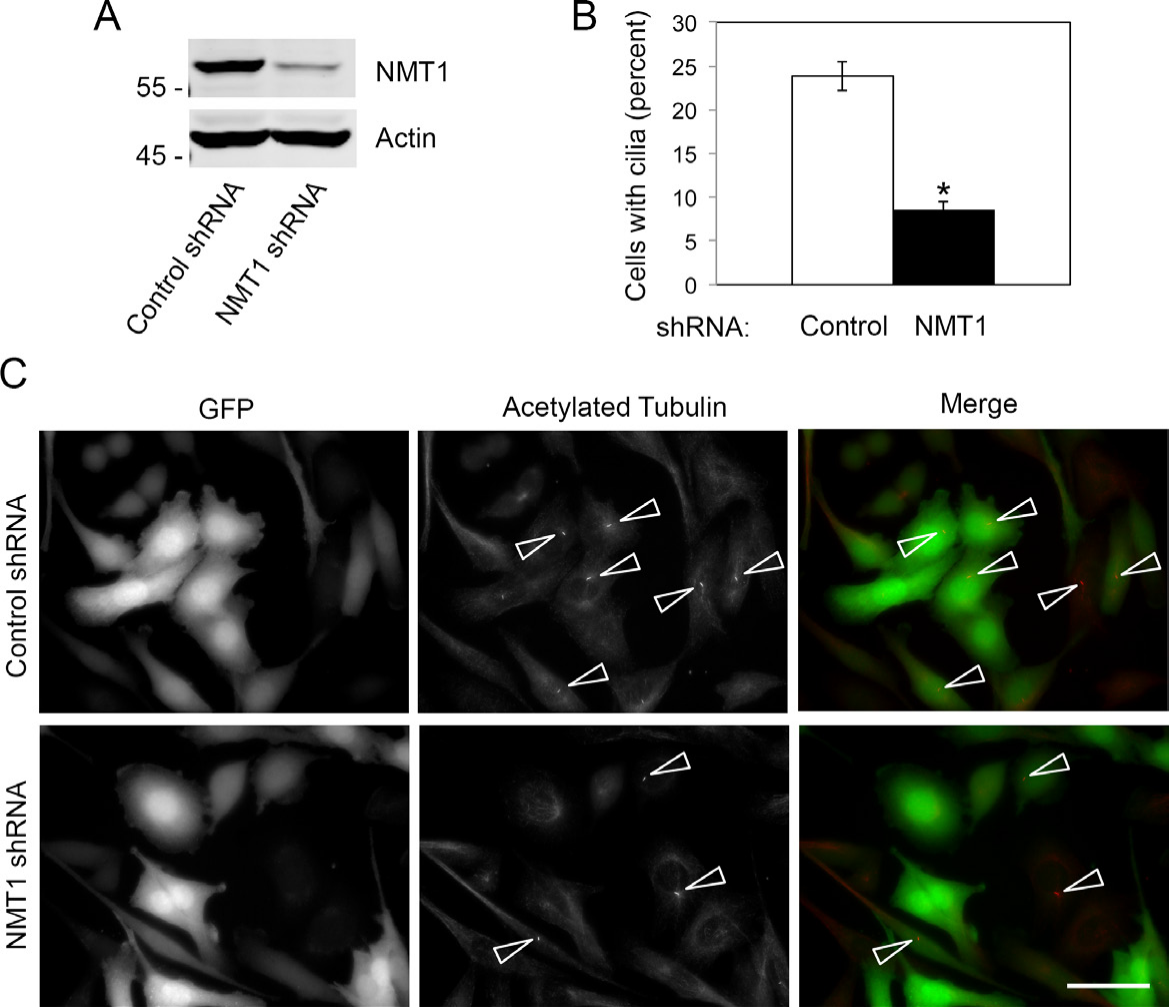
NMT1 is necessary for cilia formation in Pan02 cells (**A**) Pan02 lines expressing control non-targeting shRNA or NMT1 shRNA were cultured for 20 h and processed for detection of cilia by acetylated tubulin staining. (A) Percent of cells forming cilia was quantified in at least 250 cells per condition in three independent experiments. The graph shows average ± S.E.M. for the percent of cells forming cilia in a representative experiment out of three independent experiments. (*) *p* < 0.0001 (Student's *t*-test). (**B**) Protein extracts from control non-targeting shRNA or NMT1 shRNA Pan02 cells were processed for electrophoresis and immunoblotting with NMT1 and Actin antibodies. (**C**) Representative images of cilia (arrowheads) in Pan02 lines expressing control non-targeting shRNA or NMT1 shRNA. GFP is a marker of shRNA expression. Bar, 10 μm.

To better understand the molecular mechanism mediating the effect of tris DBA on cilia formation, we analyzed whether NMT1, the main target of this compound, affected cilia formation in Pan02 cells. We used lentiviral infection to generate Pan02 cell lines stably expressing control non-targeting shRNA or NMT1 shRNA along with GFP as a marker of infection (Figure 6A, 6C). Both cell lines were cultured for 6h in the presence or absence of tris DBA and processed for cilia detection. Importantly, silencing of NMT1 decreased cilia formation in Pan02 cells to a level that was comparable to the effect of tris DBA (Figure 6B and Figure 4A). This result indicated that the effect of tris DBA in cilia formation is mediated through NMT1. Furthermore, these data suggest that NMT1 is a novel regulator of cilia.

**Figure 7:**
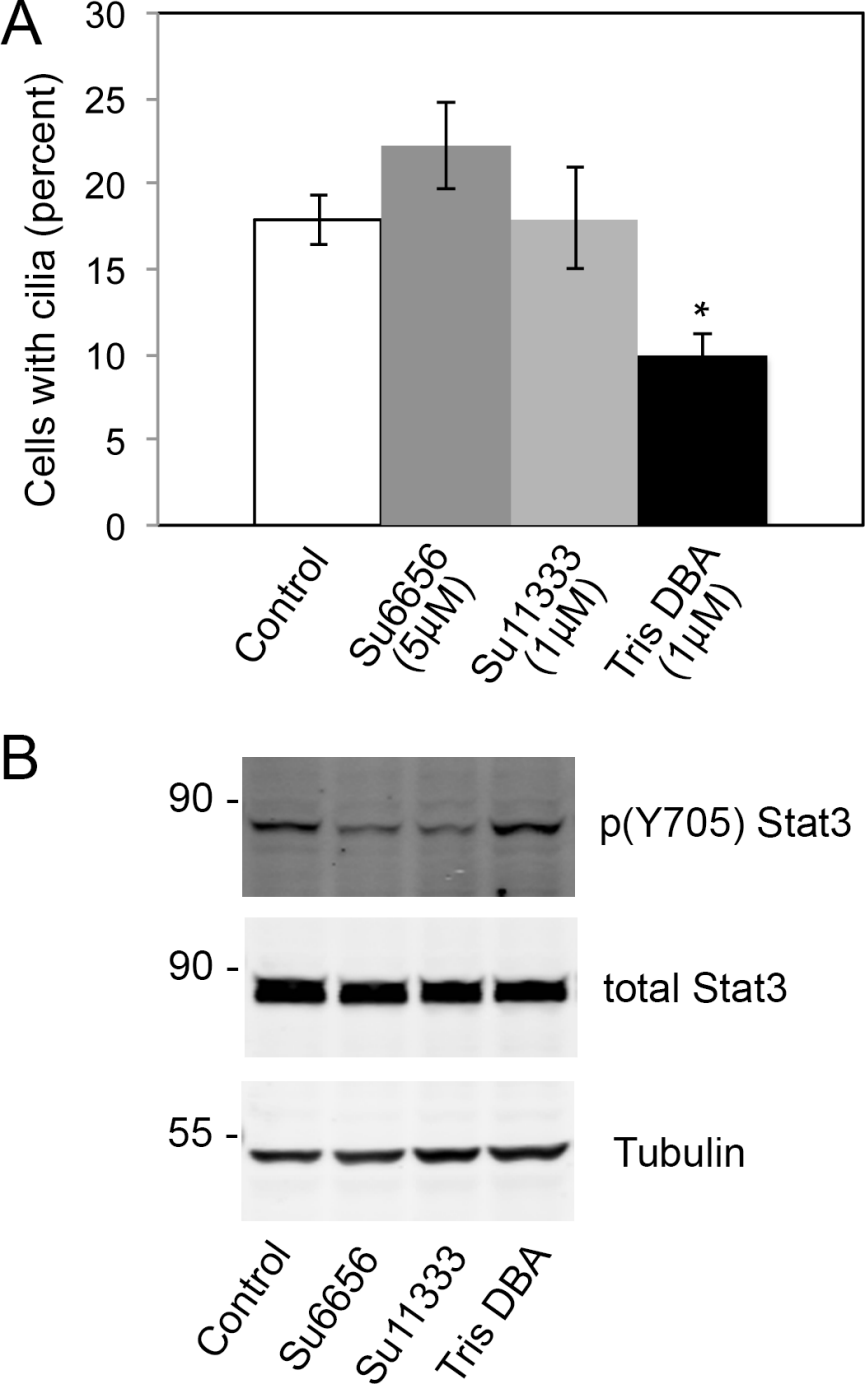
SFK activity is not required for cilia formation in Pan02 cells (**A**) Pan02 cells were treated tris-DBA (1 μM) or the SFK inhibitors Su6656 (5 μM) or Su11333 (1 μM) for 6 hours. Cells were stained with acetylated tubulin to detect cilia. Percent of cells forming cilia was quantified in at least 200 cells per condition in three independent experiments and represented as average ± S.E.M. (*) *p* < 0.001 (Student's *t*-test). (**B**) Pan02 cells were treated with tris-DBA (1 μM) or the SFK inhibitors Su6656 (5 μM) or Su11333 (1 μM) for 6 hours. Cell extracts were processed for immunoblotting with p(Y705) Stat3, total Stat3 and Tubulin antibodies to assess the effectiveness of SFK inhibition.

Since NMT1 affects the myristoylation of SFK, we analyze whether SFKs were the target of NMT1 in cilia formation. We used the SFK inhibitor SU6656 and its derivative SU11333 to probe for an effect of SFK activity in cilia formation. We incubated Pan02 cells with these inhibitors or vehicle control and assay for cilia formation 6 h after treatment (Figure 7). SFK inhibition was verified by analyzing the tyrosine phosphorylation levels of Stat3 on the SFK-specific residue 705 (Figure 7B). Inhibition of SFKs in Pan02 cells did not affect cilia formation (Figure 7A), indicating that NMT1 regulated cilia through effects on a substrate other than SFKs.

In summary, we show here that the NMT1 small molecule inhibitor tris DBA significantly decreased pancreatic primary tumor growth and metastasis of Pan02 cell in an orthotopic model *in vivo*. Tris DBA affected pancreatic cancer cell proliferation and colony formation *in vitro*, which could explain the reduction in primary tumor growth *in vivo*. Importantly, tris DBA decreased pancreatic cancer cell migration in response to a gradient of soluble factors (chemotaxis), at least in part by decreasing the ability of cancer cells to elaborate cilia in an NMT1-dependent and SFK-independent manner. The effect of tris DBA on cilia formation and chemotaxis could explain the effectiveness of this compound on diminishing metastasis *in vivo*. Finally, we described NMT1 as a novel regulator of cilia formation and tris DBA as a novel inhibitor of cilia formation.

## DISCUSSION

Pancreatic carcinoma remains one of the most intractable solid tumors, with 5 year survival rates remaining around 20%. The most widely used therapeutic is gemcitabine, although other combinations such as gemicitabine/erlotinib and folfirinox may demonstrate increased survival. Even with these combinations, pancreatic carcinoma is usually highly lethal. Both intrinsic and extrinsic factors contribute to the poor response of pancreatic carcinoma to chemotherapy and radiation.

Intrinsic factors include the oncogene and tumor suppressor profile of pancreatic adenocarcinoma. The most common mutation in pancreatic carcinoma is that of Kras, and Kras is mutated in a number of other epithelial tumors, such as colon and non-small cell lung carcinoma. No specific inhibitor of Kras has been discovered, and Kras is often considered “undruggable”. Other common mutations in pancreatic carcinoma include mutation in p53, loss of expression of p16ink4a, and loss of the TGF beta signaling molecule SMAD4/dpc4 (deleted in pancreatic cancer 4) [1, 13–15]. Epidermal growth factor (EGF) is upregulated in approximately 60% of adenocarcinomas of the pancreas, providing a rationale for the use of EGFR inhibitors in pancreatic carcinoma [16, 17].

Extrinsic factors include anatomic location and desmoplastic stroma. The stroma has been widely recognized to contribute to resistance of pancreatic carcinoma to chemotherapy. Attempts to alleviate desmoplasia to augment chemotherapy development have been mixed. Pancreatic epithelial cells secrete the ligand sonic hedgehog (Shh), which stimulates desmoplasia [5]. In a genetic mouse model of pancreatic carcinoma, treatment with a Shh inhibitor, IPI-926, led to decreased desmoplasia and increased efficacy of gemcitabine [18]. As of this date, combination vismodegib and gemcitabine have not yielded increased progression free or overall survival [4].

Here we demonstrated that tris DBA, an inhibitor of NMT1 is effective at reducing growth and metastasis of pancreatic cancer *in vivo*, indicating that NMT1 may be a novel therapeutic target in pancreatic cancer. Heme oxygen mediated carbon monoxide release and direct releasers of carbon monoxide have been shown to inhibit the growth of pancreatic carcinoma xenografts *in vivo* and block the phosphorylation of VEGFR2 [19]. Further study is required to determine whether the potent *in vivo* effects of Tris DBA palladium are mediated in part through carbon monoxide generation.

Tris DBA inhibited the growth of Pan02 primary tumors *in vivo* and had a striking effect in decreasing the formation of metastatic lesions. The effect in primary tumor growth was consistent with reduced proliferation and colony forming ability of pancreatic cancer cells treated with tris DBA *in vitro*. Interestingly, the inhibitory effect of tris DBA on the metastatic ability of Pan02 cells is likely mediated through the inhibition of directed cell migration, which facilitates intravasation, the step preceding metastatic dissemination. Directed cancer cell migration towards a soluble extracellular factor (chemotaxis) is often modeled *in vitro* by using a gradient of FBS to mimic the growth factor or cytokine gradients to which cancer cells are exposed *in vivo*. Using this assay, we uncovered an inhibitory effect of tris DBA in directed migration (chemotaxis), which was in contrast with the lack of effect of this compound in non-directed migration (chemokinesis). Chemotaxis is comprised of chemosensing, polarization and locomotion [11]. Chemosensing and polarization are specific to chemotaxis, whereas chemokinesis and chemotaxis both result in locomotion. The specific effect of tris DBA in chemotaxis but not in chemokinesis suggested that locomotion was not directly impaired by this compound, in agreement with the lack of effect of tris DBA on the general appearance of the actin cytoskeleton or in the formation of focal adhesions detected with FAK staining. Cilia are microtubule-based structures that transduce extracellular signals to regulate multiple cellular functions including cell migration [12]. Cilia regulate directed migration of fibroblasts in response to PDGF-aa [20]. Furthermore, the formation of cilia is necessary for Hedhehog and Wnt signaling [21]; which are known regulators of cell migration. The formation of cilia by pancreatic cancer cells is impaired by oncogenic k-Ras in mice [22]. However, we detect robust cilia formation in Pan02 and PANC1 cells, suggesting that these pancreatic cancer cells are cilia-dependent. Recent studies indicate that impaired cilia formation is not a general phenomenon in pancreatic cancer, where the formation of cilia in a subset of lesions correlates with poor prognosis in clinical samples from pancreatic ductal adenocarcinoma patients [10]. Furthermore, recent evidence indicates that cilia disruption may not be a common feature in cancer, with some cancers actually relying in ciliary signaling [12].

Our data indicate that Pan02 pancreatic cancer cells depend on cilia formation for directional migration towards soluble factors *in vitro*. Importantly, we describe here that cilia are regulated by NMT1 and, therefore, a target for the NMT1 inhibitor tris DBA. This suggests a mechanism by which this compound decreases chemotaxis *in vitro* and diminishes metastasis *in vivo*. SFKs members including c-Src are associated with tumor progression and metastasis in a variety of cancers, including pancreatic cancer [7]. SFK member are known NMT1 targets [23], and their localization and function is regulated by N-terminal myristoylation. The SKF inhibitor SU6656 [24] and its derivative SU11333 [25] were used to probe for a role of SFKs downstream NMT1 in cilia formation. The lack of effect of SFK inhibitors in this context indicated that NMT1 was modulating cilia formation in Pan02 cells in a SFK-independent manner. These results are in agreement with an inhibitory role of increased Src activity in ciliogenesis in mouse embryonic fibroblasts [26]. It is possible that tris DBA decreases N-myristoylation and ciliary membrane localization of additional proteins that are important for cilia formation. Indeed, myristoylation targets proteins to the eukaryotic ciliary membrane [27] and several ciliary proteins are myristoylated, including Cystin [28] and the ciliopathy protein nephrocystin-3 or NPHP3 [29]. In addition, many ciliary proteins identified by proteomic analysis are predicted to be N-myristoylated [30]. Further research will be necessary to determine the specific targets of tris DBA at cilia as well as the signaling mechanisms that regulate cilia-dependent directed migration in pancreatic cancer cells. Several studies suggest that NMT1 activity is important for the progression of different malignancies including breast cancer [31], oral squamous cell carcinoma [32], colon carcinoma [33] and gallblader cancer [34]. Our findings suggest that NMT1 is a novel therapeutic target in pancreatic cancer and warrants further investigation of tris DBA as a potential novel treatment for pancreatic carcinoma.

## MATERIALS AND METHODS

### Cell lines

The murine pancreatic cancer cell line Pan02 (Panc02) was obtained from National Cancer Institute and confirmed to be pathogen-free before use. PANC1 human carcinoma cells were obtained from ATCC. Cells were cultured in DMEM (Invitrogen) with 10% fetal bovine serum and maintained at 37°C in a humidified incubator with 5% CO_2_ and 95% air.

### Compounds

Tris DBA palladium was obtained from Sigma-Aldrich (Catalogue number 328774), and was dissolved in ethanol for *in vitro* studies. The SFK inhibitors SU6656 and SU11333 (prepared in DMSO) were a generous gift from Dr. Sara Courtneidge (OHSU, Portland, OR).

### Antibodies

The following primary antibodies were used: anti-p(S473) Akt, anti-Akt (total), anti-p(T202/Y204) Erk1/2, anti Erk1/2 (total), anti-p(Thr389) p70 S6K, anti-FoxM1, anti-p(S705) Stat3, anti-Stat3 (total), anti-beta Tubulin (clone 13E5), all from Cell Signaling Technology. Anti-NMT1 was from Proteintech (11546-1-AP), anti-acetylated tubulin antibody (clone 6-11B-1), from Sigma, anti-FAK antibody (clone 4.47) from EMD Millipore, and anti-b-actin from Santa Cruz.

### Western blot

Protein lysates were obtained using RIPA buffer containing protease and phosphatase inhibitors. Protein concentration was quantified and lysates denatured with β-mercaptoethanol and heat at 95°C for 5 minutes. After SDS-PAGE, proteins were transferred to PVDF membranes. The blots were then incubated at room temperature for 1 hour in 5% non-fat dry milk in tris-Buffered Saline and Tween 20 (TBST) and blotted overnight at 4°C with the corresponding primary antibodies. Afterwards they were washed 3× with TBST and incubated at room temperature for 1 hour with the corresponding secondary antibody. Detection was achieved using the Bio-Rad chemi-dock and imaging software, Image Lab 4.0 software or an infrared imaging system (Odyssey; LI-COR Biosciences).

### Proliferation assay

5 × 10^4^ Pan02 cells per well were plated in a 24-well plate. Cells were washed with 1× PBS and treated in quadruplicates for 24 hours. The treatment was then removed and the cells were washed with PBS, trypsinized and counted. Cells were counted using a Beckman Z-1 Coulter Counter.

### Colony forming assay

500 cells per well were seeded in 6-well plates and treated with the indicated concentrations of tris DBA palladium in triplicates 24 h after plating. Plates were incubated for 6 days in the presence of compound and processed for crystal violet staining. Plates were scanned and the number of colonies manually counted.

### Animal studies

All animals were housed in pathogen-free facility with access to food and water ad libitum. Experiments were approved and performed in accordance with the Institutional Animal Care and Use Committee at the University of Texas Southwestern Medical Center. C57BL/6 mice were purchased from on campus supplier. Six to eight week old male mice, in a group of five for vehicle control and a group of seven for tris DBA palladium, were injected orthotopically with 5 × 10^5^ Pan02 cells and randomized 1 week after tumor cell injection to receive 3 weekly i.p. injections of vehicle (coconut oil 100 μL), or tris DBA palladium (6.4 mg/kg). Mice were sacrificed when moribund after 4 weeks. At the time of sacrifice, orthotopic tumors were removed in bloc to determine primary tumor burden. Gross metastatic events were identified through visual inspection of lymph node basins, liver, peritoneum, diaphragm and spleen. Samples were fixed in 10% formalin or snap frozen in liquid nitrogen for further studies. Data were analyzed using GraphPad software (GraphPad Software, San Diego, CA). Results are expressed as mean +/− SEM. Data were analyzed by one way ANOVA with differences considered significant at *p* < 0.05.

### Trans-well migration assay

We used 8 μm pore polycarbonate membranes (Corning). Cells were seeded in triplicate (1 × 10^5^ per insert) in the presence or absence of Tris-DBA (1 μM) in 500 μl of medium containing 1% (for chemokinesis assay) or 10% FBS (for chemotaxis assay). The lower chamber was filled with 750 μl of medium containing 10% FBS. After 6 h at 37^°^C, inserts were washed in PBS and cells in the upper side of the membrane removed with wet cotton-tipped swaps. Inserts were fixed in 4% paraformaldehyde for 15 minutes, followed by one PBS wash and incubation in a crystal violet solution (0.1% crystal violet in 30% methanol) for 30 minutes. Inserts were washed extensively with distilled water and let dry. Migrated cells were quantified under a microscope (inverted CKX31 Olympus) in at least six random fields per insert. Pictures were taken with an inverted IX81 Olympus Wide Field and Fluorescence Microscope (using an UPlan Apo 10× objective) equipped with a color cooled CCD Spot RT3 camera (Diagnostic Instruments Inc.) and MetaMorph software (UIC dev. of Molecular Devices). Color digital images were inverted using Adobe Photoshop software (Adobe Inc).

### Crystal violet assay

Cells were seeded in triplicate in 96-well tissue culture plates (2 × 10^4^ per well), in the presence or absence of tris-DBA (1 μM) in 500 μl of medium containing 1% or 10% FBS. After 6 h cells were stained with crystal violet solution (0.1% crystal violet in 30% methanol) for 30 minutes, washed extensively with distilled water and let dry. Stain was eluted in 150 μl of 1% SDS in water for 30 minutes. An aliquot of 100 μl per well was transferred to a clean 96-well plate and absorbance was measured at 570 nm using a microplate reader (EL×800; BioTek Instruments Inc.).

### Detection and quantification of cilia in cells

Pan02 cells (4 × 10^4^ per well) were seeded on 12-well plates containing 18 mm diameter glass coverslips in the presence or absence of tris DBA (1 μM) in medium containing 10% FBS and incubated at 37^°^C for 6 or 20 h. Cells were washed with PBS and fix in 4% paraformaldehyde for 10 minutes. After 30 minutes of blocking with PBS containing 0.1% Triton X-100 and 3% BSA, cells were incubated with a 1:1000 dilution of anti-acetylated tubulin antibody in PBS containing 0.1% Triton X-100 and 0.3% BSA for 2 hours at RT. Cells were washed with PBS containing 0.1% Triton X-100 and incubated with a 1:500 dilution of Alexa 488-conjugated goat anti-mouse antibody (Invitrogen) and 1:500 dilution of Alexa 568-conjugated phalloidin (Invitrogen) in PBS containing 0.1% Triton X-100 and 0.3% BSA for 1 hour at RT. Cells were washed and mount in medium containing DAPI (Vector Labs.). Images were obtained in a fluorescent microscope (Axioplan 2; Carl Zeiss) equipped with a charge-coupled device camera (AxioCam HRm; Carl Zeiss) and AxioVision software (Carl Zeiss). Quantification was performed on 10 randomly acquired images (representing around 200 cells per coverslip) obtained at 20× magnification.

### Detection and quantification of cilia in tumor sections

Tumors were fixed in formalin, paraffin-embedded and sectioned. Sections were de-paraffined using Xylene and Ethanol and re-hydrated in distilled water and PBS. An acidic antigen retrieval solution was used before incubation of samples with the primary antibody (1:2,000) overnight at 4^°^C. After extensive washing, samples were incubated with Alexa 488-conjugated anti-mouse antibody (1:400) for 2 hours. Sections were mounted in Vectashield-containg DAPI (Vector Labs). Images were obtained in a fluorescent microscope (Axioplan 2; Carl Zeiss) equipped with a charge-coupled device camera (AxioCam HRm; Carl Zeiss) and AxioVision software (Carl Zeiss). Quantification was performed on 15 randomly acquired images (representing around 90 cells per field) obtained at 40× magnification. Number of nuclei per section was used for normalization.

### Generation of NMT1 deficient Pan02 cell lines

Pan02 cells were infected with lentiviral particles generated from pLKO.1 puro CMV-turbo GFP constructs expressing either control non-targeting shRNA (SHC003, Sigma) or NMT1 shRNA (SHCLNV-NM_008707, TRCN0000055163, Sigma). Cells were selected with Puromycin (5 μg/ml) for 7 to 10 days. NMT1 knockdown was verified by immunoblotting using an anti-NMT1 antibody.

## SUPPLEMENTARY MATERIALS FIGURES


