# Supplementary material for: Tris DBA palladium is highly effective against growth and metastasis of pancreatic cancer in an orthotopic model
Source: Oncotarget. 2016 Jul 9;7(32):51569–80. doi: 10.18632/oncotarget.10514 (PMC5239497; doi:10.18632/oncotarget.10514)
Supplement: Supplementary file 1 [file oncotarget-07-51569-s001.pdf]

Tris DBA palladium is highly effective against growth and metastasis of pancreatic cancer in an orthotopic model

Supplementary Materials

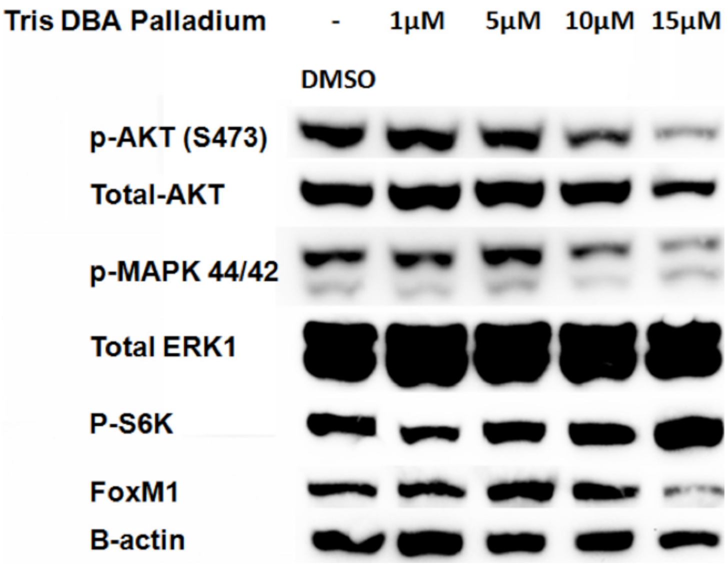

Supplementary Figure S1: Effect of tris DBA in the activation of different signaling pathways. Pan02 cells were treated with the indicated concentrations of tris DBA for 24 h. Cells were lysed and analyzed by western blotting using the indicated antibodies. B-actin was used as loading control.

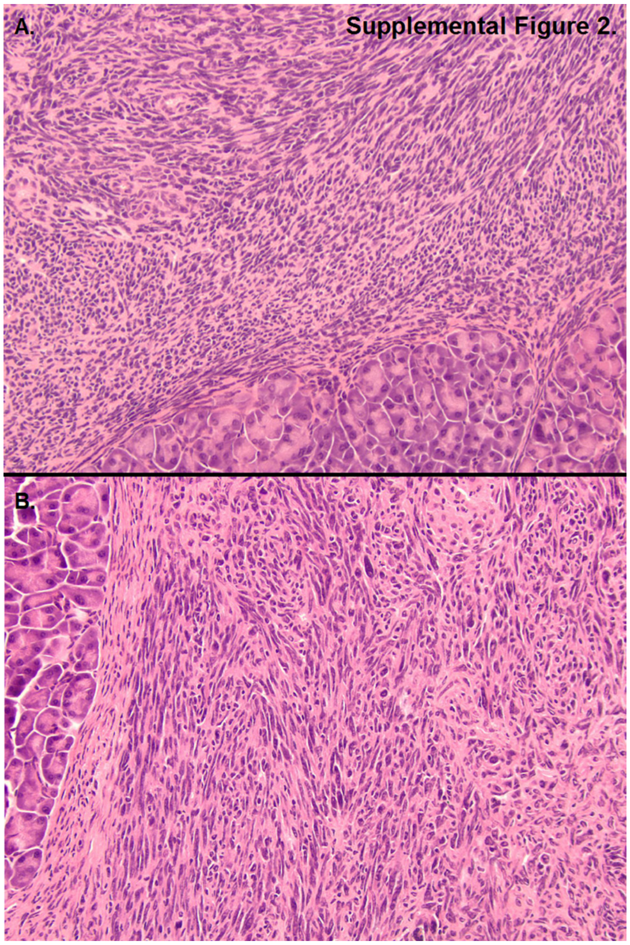

Supplementary Figure S2: Histological hematoxylin and eosin (H&E) staining on Pan02 tumors. (**A**) Control group. (**B**) Tris DBA group.

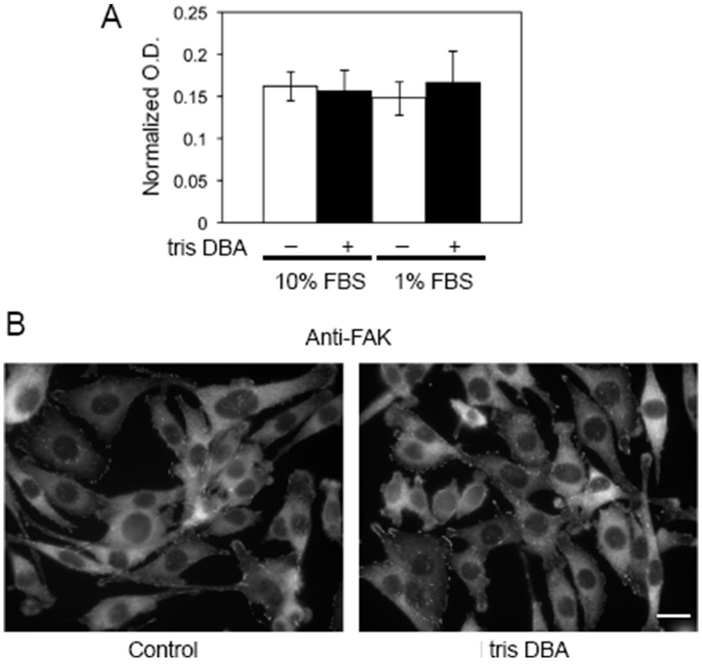

Supplementary Figure S3: Plating efficiency or focal adhesion formation are not affected by treatment of Pan02 cells with tris DBA. (**A**) To assess the plating efficiency of Pan02 cells in either 1% or 10% FBS in the presence or absence of tris-DBA (1 µM), cells were cultured for 6 h under the indicated conditions and stained with crystal violet. Cell number was quantified by measuring absorbance, and represented as average ± S.D. of optical density (O.D.) values. (**B**) Cells were cultured in the presence or absence of tris DBA (1 µM) for 20 h and stained for FAK. Representative images are shown. Bar, 5 µm.
